# NMR spectroscopy reveals the presence and association of lipids and keratin in adhesive gecko setae

**DOI:** 10.1038/srep09594

**Published:** 2015-04-22

**Authors:** Dharamdeep Jain, Alyssa Y. Stark, Peter H. Niewiarowski, Toshikazu Miyoshi, Ali Dhinojwala

**Affiliations:** 1 Department of Polymer Science, The University of Akron, Akron, OH 44325-3909, USA; 2 Department of Biology, Integrated Bioscience Program, The University of Akron, Akron, OH 44325-3908, USA

## Abstract

Lipid and protein aggregates are one of the fundamental materials of biological systems. Examples include cell membranes, insect cuticle, vertebrate epidermis, feathers, hair and adhesive structures known as ‘setae’ on gecko toes. Until recently gecko setae were assumed to be composed entirely of keratin, but analysis of footprints left behind by geckos walking on surfaces revealed that setae include various kinds of lipids. However, the arrangement and molecular-level behavior of lipids and keratin in the setae is still not known. In the present study we demonstrate, for the first time, the use of Nuclear Magnetic Resonance (NMR) spectroscopy techniques to confirm the presence of lipids and investigate their association with keratin in ‘pristine' sheds, or natural molts of the adhesive toe pad and non-adhesive regions of the skin. Analysis was also carried on the sheds after they were ‘delipidized’ to remove surface lipids. Our results show a distribution of similar lipids in both the skin and toe shed but with different dynamics at a molecular level. The present study can help us understand the gecko system both biologically and for design of synthetic adhesives, but the findings may be relevant to the characteristics of lipid-protein interactions in other biological systems.

Lipids form an integral part of various biological systems^1^,^2^. One of the key examples, the epidermis (mammalian, reptilian or avian), consists of lipids surrounding dead keratinous cells in the upper region of the skin known as stratum corneum^3^,^4^. Lipids help in maintaining physical resistance and serve as an epidermal water barrier^5^,^6^. Besides acting as a skin barrier, lipids have been associated with a variety of biological attachment strategies such as the hairy structures on the chitin-based cuticle of insects^7^,^8^,^9^, podia in sea stars^10^ and cement secretions in barnacles^11^. Other roles include their presence as a protective coating in dragline silk in spiders^12^, as well as in self-assembly of the proteins in mussel byssal threads^13^. Thus there is increasing interest in lipids from multiple fields, but little work has been focused specifically on them.

One system of great interest recently has become the gecko adhesive system, where lipids have also been confirmed in the small hair-like adhesive structures^14^ and in invisible footprint residue that is left behind as they walk^15^. In general, geckos have historically been known for their popular ‘smart’ keratinous fibrillar adhesive^16^, which is comprised of highly organized similarly oriented and uniformly distributed microscopic hairy structures known as setae (Figure 1a), which further branch at the tips into spatula^16^,^17^,^18^. In addition to the numerous ultrastructural, immunological and histological analyses^19^,^20^,^21^,^22^,^23^, the use of Microbeam X-ray diffraction and Raman spectroscopy^24^ have confirmed that the main constituent of setae is stiff keratinous material. Keratin is a fibrous and structural protein that finds a prominent role in mammals (hair, wool, horn, fur, nail and skin), reptiles (scales and claws), birds (feather, beak and claw) and fish (teeth and slime)^25^,^26^. Various biochemical analyses^19^,^20^,^21^,^22^,^23^ suggest that during development, gecko setae incorporate keratin at their base, which is further deposited into long bundles oriented along main axis of setae. The adhesive setae consists of specific keratin associated beta proteins (KAbetaPs) and various forms of α-keratin^19^,^20^,^21^,^22^,^23^. The keratin-based adhesive setae have a high elastic modulus, which is likely used to maintain the robustness of the setal structure during repeated attachment and detachment^16^. However, the recent discovery of phospholipid footprints, and their potential to be at the adhesive contact interface^15^ has puzzled many and given a new dimension to existing keratin-based models of the gecko adhesive system.

Nano Assisted Laser Desorption Ionization (NALDI) mass spectrometry measurements confirmed the presence of the phospholipid ‘dipalmitoylphosphatidylcholine’ (DPPC) (Figure 2) in the traces of the footprint residue, while Sum Frequency Generation (SFG) spectroscopy showed the presence of hydrophobic methyl and methylene groups at the contact interface between the gecko toe pad and substrate^15^. Additionally, histochemical studies have shown the presence of lipids packed with the keratin material in the adhesive setae^14^. The presence of lipids and their potential association with the keratin in the gecko setae calls into question their possible function in self-assembly of keratin bundles^2^, adhesion (dry and wet)^27^,^28^,^29^, self-cleaning^30^,^31^, superhydrophobicity^32^, ductility and wear of the system^12^. Since the setal structure is a combination of keratin and lipids, one of the key questions is how the keratin and lipid components are associated in the setal structure. Hence, there is a need to study the assembly of these constituent materials, identify the interaction between them, and understand the structure and dynamics of this essential feature at a molecular level, all of which has been severely lacking in gecko adhesion literature.

Motivated by Solid-State NMR studies of α-keratin^33^,^34^,^35^,^36^,^37^,^38^,^39^,^40^ focused on relating the macro properties of the material with the structure and dynamics of the molecules^41^, we report the first ever Solid-State NMR (Supplementary Text S1) analysis done on the molts (sheds) of the Tokay gecko (*Gekko gecko*)(Supplementary Figure 1). In addition to the rows of setae in the toe pad shed (~ 65–70% of the shed, Supplementary Text S2), the molt is also comprised of several other layers of epidermis^22^. In order to confirm that the NMR signal is dominated by setae, skin sheds from the non-adhesive epidermis (Figure 1c) have also been studied to present a comparative view. Based on the finding of phospholipid footprint residue^15^, the current work hypothesizes that (a) the lipids are present in the setae, and (b) the lipids in the setae are loosely bound and are mobile at the NMR timescale. To test this we first removed unbound lipids off the sheds (chloroform methanol exposure)^42^ and used Solution State NMR^43^ and TLC^44^,^45^ to analyze and confirm the presence of lipids in both the toe and skin shed. Second, we used Solid-State NMR based Magic Angle Spinning (MAS) techniques (Cross Polarization (CP/MAS), Direct Polarization (DP/MAS) and Proton (^1^H/MAS), Supplementary Text S1) to establish the keratin and lipid related peaks as well as to probe the dynamic behavior of the two components present in the shed. Our results help to clarify the lipid-keratin association in both the adhesive gecko setae and non-adhesive skin, as well as provide insight to improve fabrication designs for synthetic adhesives.

## Results

### Delipidization

**Figure 1:**
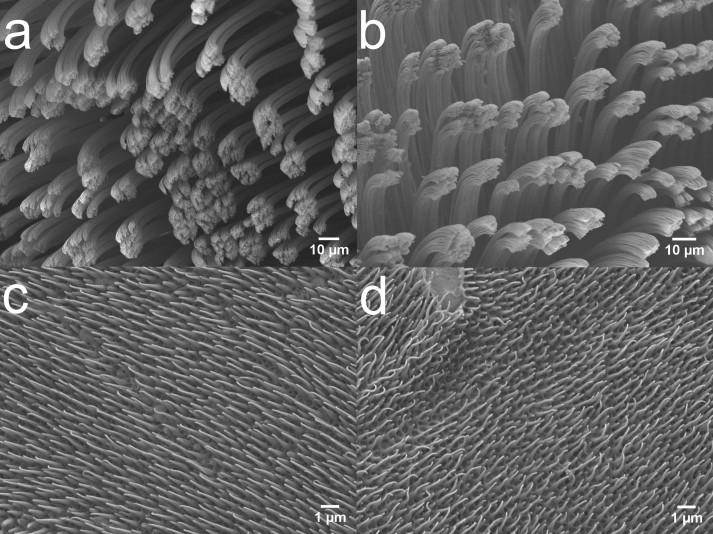
Pristine and Delipidized Sheds. Scanning electron micrographs (SEM) for pristine (a) and delipidized (b) Toe sheds, showing the adhesive hairy features known as ‘setae’ and pristine (c) and delipidized (d) Skin sheds, showing the ‘spinulae’ structures respectively. These structures remain unaffected after the lipid extraction.

The hydrophobic lipid footprint residue^15^ is anticipated to be unbound lipid associated with the setal structure. To test this hypothesis, we carried out the lipid extraction technique described by Swartzendruber et al.^42^ to remove the unbound lipids. The technique has been used previously to extract lipids from lizard skin^46^ the results of which match our current lipid extracts from skin (~10–12 wt% of the mass of the sheds). Interestingly, the amount of extractable lipid material from the toe shed was found to be around ~ 8–10 wt%, slightly less than the skin. Keratin is insoluble in organic solvents^26^ hence, we do not believe keratin is being removed by the treatment. Furthermore, when investigating the pristine and delipidized samples, we did not see any obvious change in morphology of the setae (Figure 1b) and spinulae (Figure 1d).

### Analysis of lipid extract

Standard lipid characterization techniques such as Thin Layer Chromatography (TLC) and Solution-State NMR were used to analyze the lipid extracts from toe and non-adhesive skin sheds. Table 1 lists the TLC results (R_f_ values of the lipids) using primuline as the detection agent. Lipids including phospholipids such as sphingomyelin (SM), phosphatidylcholine (PC), and phosphatidylethanolamine (PE) as well as non-polar lipids (probably glycerides, fatty acids and cholesterol) were seen in the toe and skin extracts. In addition to the R_f_ values available in literature^47^, the presence of SM and PC was confirmed by comparing with standard sample spots. PE was confirmed by spraying the plate with ninhydrin. Spots were also visualized with 40% sulphuric acid spray.

In addition to TLC, we used Solution-State NMR to further probe the extracted lipid solution. Standard samples of PC and SM show peaks at −0.64 ppm and −0.04 ppm respectively (Figure 3a). The toe shed extract shows peaks at −0.64 ppm and −0.08 ppm (Figure 3b), which confirms the presence of phospholipids PC and PE (as detected in TLC)^43^. The SM peak lies near the PE peak and we anticipate it to lie within the shoulder of the broad PE peak (Figure 3b). Similar peaks are seen in the skin shed extract (Supplementary Figure S2). Clearly, non-polar lipids cannot be detected with this technique due to the absence of a phosphorus moiety in their structure. In general, the reptilian epidermis is associated with non-polar lipids such as free fatty acids, cholesterol and triglycerides (as shown in the TLC results) as well as polar lipids like phospholipids and sphingomyelin (TLC and NMR results)^46^, although it is possible that there are other lipid species present in the setae. Our work here however is the first to report that in addition to the phospholipids detected in the NALDI study^15^ (PC and SM), other lipid types are also in the adhesive toe pad extract. We confirmed that the delipidization treatment did not remove keratin from the sheds using ^1^H NMR (Figure 3c). Peaks at 0.8 ppm (ωCH_3_) and 1.1 ppm ((CH_2_)_n_) in addition to other lipid-based peaks (inset Figure 3c) further confirmed the presence of lipids in the extract^48^. Proteins usually show a crowd of peaks in the range of 1–5 ppm in ^1^H NMR^49^, which is absent in the lipid extract spectra, suggesting that keratin was not removed from the toe or skin shed during the delipidization treatment and thus will did not affect our analysis.

### Solid State NMR

#### (a) Cross Polarization/Magic Angle Spinning (CP/MAS)

Figure 4 shows the ^13^C CP/MAS spectra of pristine and delipidized toe sheds for *Gekko gecko*. Since this Solid-State NMR technique is sensitive to molecules with slow dynamics^39^, the keratin dominated spectra reveals that the amino acids forming the structural protein keratin are rigid at the frequency less than ~10 kHz^39^. Peaks were assigned (Supplementary Table S1) by taking into consideration previous studies on keratin-based systems using solid-state NMR^33^,^34^,^35^,^36^,^37^,^38^,^39^, as well as biochemical results for amino acids specific to the proteins constituting setae and skin^20^,^50^. The spectra can be divided into four regions: carbonyl, aromatic, C_α_ and aliphatic^33^,^35^,^36^,^37^. The carbonyl region shows a distinctive peak including signatures from the carbonyl backbone present in amino acids comprising the keratin. The aromatic region shows peaks from amino acids such as tyrosine and phenylalanine. The C_Z_ for arginine is the only exception which despite being aliphatic appears in the aromatic region. The broad peak between 46–60 ppm consists of C_α_ resonances from amino acids (except glycine) in keratin. The alpha carbon for glycine is conspicuous around 43 ppm^33^,^35^,^36^,^37^,^38^. The aliphatic region is dominated with signatures from cysteine, proline, isoleucine and alanine. Similar peaks are seen for the non-adhesive skin (pristine and delipidized, Supplementary Figure S3a, Supplementary Table S2)

**Figure 2:**
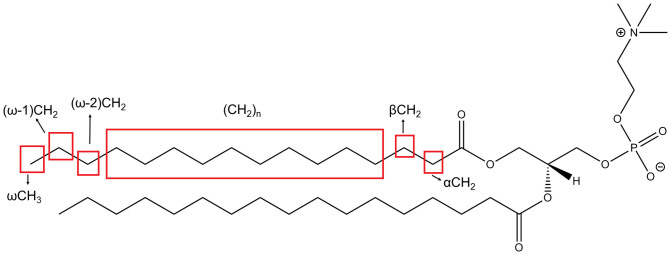
Structure of dipalmitoylphosphatidylcholine (DPPC) phospholipid. The phospholipid structure shows the positions of various methylene (CH_2_)_n_, (ω-1)CH_2_, (ω-2)CH_2_, αCH_2_ and βCH_2_ and methyl ωCH_3_ groups. These signatures are detected in the NMR experiments. The structure is shown as a model example to understand the different peaks in the NMR results.

Amidst the keratin dominated spectra, it was interesting to observe the peaks related to lipids in the aliphatic region (33 ppm, 30 ppm and 14 ppm) for toe and skin sheds (Figure 4, Supplementary Figure S3a). Such peaks have been observed previously in keratin-based systems^35^,^36^,^37^,^38^,^39^. Generally, lipids (DPPC as an example, Figure 2) show distinctive peaks at 33 ppm and 30 ppm corresponding to the CH_2_ repeating units, and another peak at 14 ppm due to the terminal methyl (ωCH_3_) in their structures^35^,^36^,^37^,^38^,^39^. The lipid peaks observed in NMR would be a contribution from both the unbound lipids as well as esterified bound lipids present in the toe or skin sheds. To confirm we were removing unbound lipids using the method described previously^42^, lipid peak intensities in delipidized sheds were observed. The reduction in the lipid peak intensities (33 ppm and 30 ppm ~ (CH_2_)_n_ and 14 ppm ~ ωCH_3_) in delipidized toe and skin sheds confirms the removal of loosely bound lipids (Figure 4, Supplementary Figure S3a). Post delipidization we do still see a small peak in lipid regions, which is likely from the esterified lipids.

#### (b) Direct Polarization/Magic Angle Spinning (DP/MAS) and ^1^H/Magic Angle Spinning (^1^H/MAS)

To assess the mobility of the lipids present in the sheds, DP/MAS (Figure 5a, Supplementary Figure S4) and ^1^H/MAS (Figure 5b, Supplementary Figure S3b) techniques were used. Sharp signals in these techniques indicate the presence of mobile molecular segments in the sample, contrary to CP/MAS (Supplementary Text S1). The majority of the signal in pristine toe shed spectra is concentrated in the aliphatic range (Figure 5a inset, 0–50 ppm), which is the lipid dominant region, indicating lipids are more mobile than the keratin in the toe sheds^39^. In addition, the carbonyl region shows a broad peak and most of the amino acid peaks seem to be absent except the broad peak in the aliphatic region and few sharp signatures in the aromatic region, again indicating that the keratin constituent is rigid compared to the mobile lipid material in the toe sheds. The sharp keratin signatures detailed above in CP/MAS (Figure 4), further confirms the rigidity of keratin and compliments the DP/MAS results. In detail, sharp lipid signatures in the DP/MAS results can be seen at 37.9 ppm, 32–33 ppm, 30.5 ppm, 25.2 ppm, 23.4 ppm and 14.7 ppm corresponding to (CH_2_)_n_, (ω-1)CH_2_, (ω-2)CH_2_, ωCH_3_, αCH_2_ and βCH_2_ respectively^39^ (Figure 5a inset, Supplementary Table S1). Upon delipidization, these prominent peaks, specifically the CH_2_ and ωCH_3_ region, reduce in intensity. Similar trends are seen in skin shed spectra, indicating lipids are mobile in the skin as well (Supplementary Figure S4).

Figure 5b shows the ^1^H MAS spectrum for pristine and delipidized toe sheds. Considering the phosphatidylcholine (DPPC) structure as an example (Figure 2). The peaks ranging from 1.1 ppm to 1.4 ppm corresponds to (-CH_2_-)_n_, (ω-1)CH_2_ and (ω-2)CH_2_; 0.7 ppm corresponds to terminal alkyl protons (ωCH_3_); and 1.8–2.5 ppm covers protons at α/β positions next to carbonyl group^51^. The broad peak seen at 3.9–4.7 ppm encompasses the alpha protons from the amino acids^52^ constituting the keratin in the toe shed. This range can also include signatures from other protons in the lipid structure^48^. The appearance of the sharp peak at 4.8 ppm riding over the broad peak may be potentially attributed to the presence of water in the sample. After the toe shed sample was delipidized, the reduction in the intensity of the lipid peaks is evident in the spectra. Again, the peak intensities for 1.1 ppm ((-CH_2_-)_n_, (ω-1)CH_2_ and (ω-2)CH_2_) and 0.7 ppm (ωCH_3_)) regions are affected by delipidization (Figure 5b), confirming the removal of unbound lipids. Similar peaks can be seen in skin shed both prior to and after removal of unbound lipids (Supplementary Figure S3b).

## Discussion

Reptilian epidermal lipids playing an important role as a barrier to water loss^3^ are primarily of two types: polar and non-polar. Polar lipids include phospholipids such as phosphatidylcholine, phosphatidylethanolamine, phosphatidylserine, phosphatidylinositol, lysophosphatidylcholine and sphingomyelin, while non-polar lipids include cholesterol, diacylglycerols, alcohols, free fatty acids, aldehydes, wax esters and sterol ester^46^. Although it was known that these lipids are present in the mesos and alpha layers of the reptilian epidermis^14^, it was surprising to detect them also in the oberhautchen layer and maturing setae being formed during regeneration cycles^14^ and in gecko footprints^15^.

**Figure 3:**
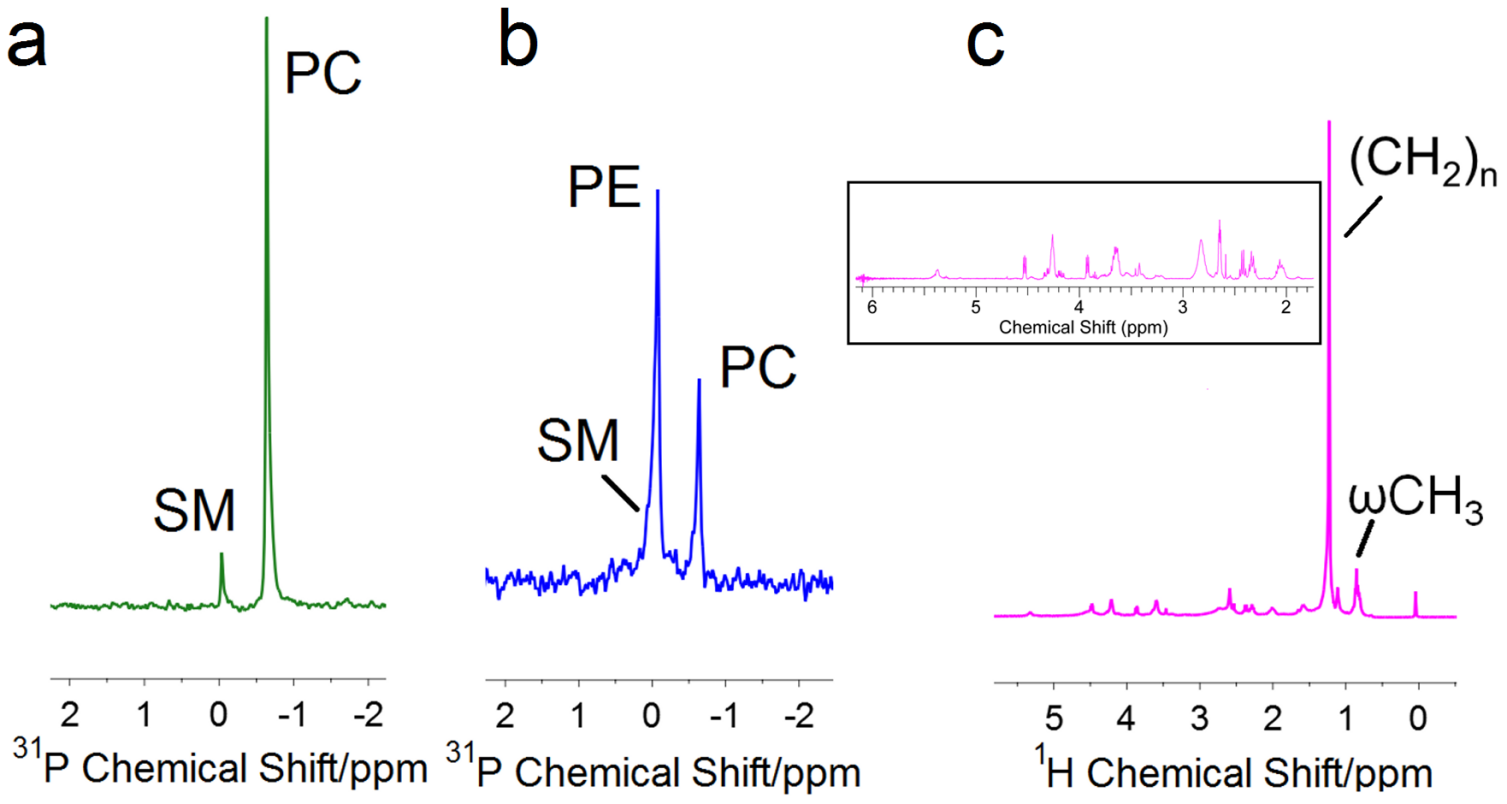
^31^P and ^1^H NMR Solution State NMR for lipid extracts from toe sheds. Figure a shows ^31^P NMR for standard phospholipids (PC and SM), figure b shows the ^31^P NMR for extracted phospholipids and figure c is the ^1^H NMR for lipid extract from toe sheds. Inset in figure c is the enlarged spectrum from 2–6 ppm.

**Figure 4:**
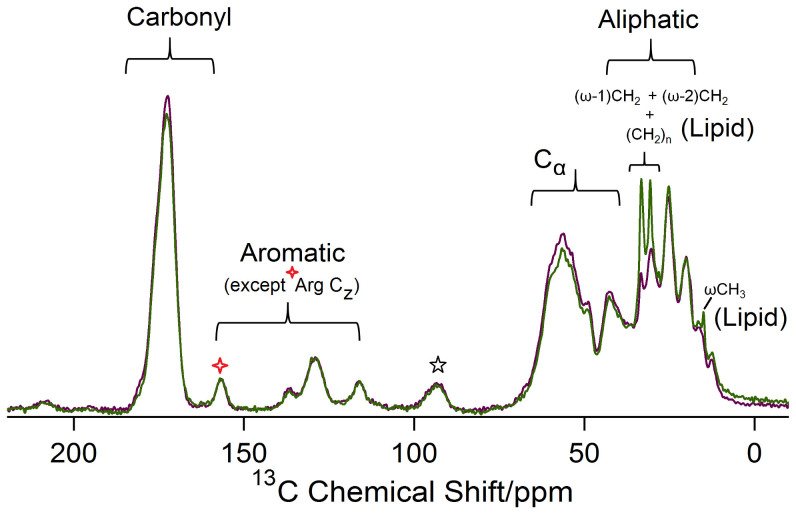
Cross Polarization/Magic Angle Spinning (CP/MAS) for Pristine and Delipidized Toe Sheds. Figure shows the CP/MAS spectra shows the pristine (green) and delipidized (purple) for toe sheds. The spectra highlights the rigid components in the sample. In addition to the aromatic Tyr C_ζ_ at 156 ppm (red star), the peak also has contribution from non-aromatic Arg C_z_. Lipid peaks occur in the aliphatic region at 33 ppm, 30 ppm [(CH_2_)_n_, (ω-1)CH_2_, (ω-2)CH_2_] and 14 ppm [ωCH_3_]. Delipidized spectrum shows the reduction in the lipid peak while the protein peaks seem mostly unaffected. The spectra are measured at MAS frequency ~6000 ± 3 Hz. Star labelled peak refers to spinning sideband.

**Figure 5:**
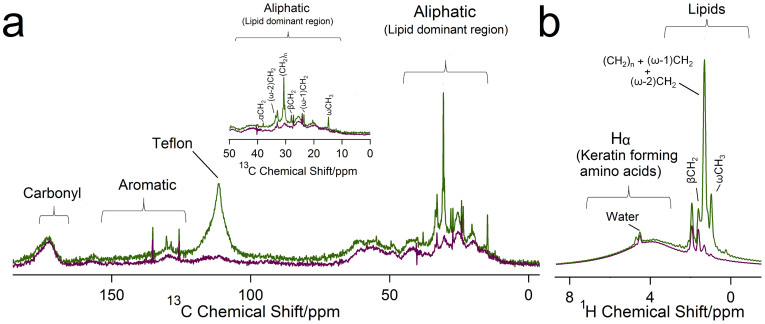
Direct Polarization Magic Angle Spinning (DP/MAS) and ^1^H Magic Angle Spinning ( ^1^H/MAS) for Pristine and Delipidized Toe Sheds. Figure a shows the DP/MAS spectrum for the pristine (green) and delipidized (purple) toe sheds. Sharp signals dominate the aliphatic region that include the lipid peaks. The inset is the enlarged aliphatic region (0–50 ppm) showing lipid signatures (CH_2_)_n_, (ω-1)CH_2_, (ω-2)CH_2_, αCH_2_, βCH_2_ and (ωCH_3_). Teflon is used as a packing material and has strong signal in direct polarization. Figure b shows the ^1^H/MAS spectra for pristine (green) and delipidized (purple) toe sheds with prominent lipid peaks at 0.6 ppm (ωCH_3_) and 1.1 ppm (CH_2_)_n_. Both techniques highlight the mobile components in the samples. The delipidized spectra (purple) confirms the removal of lipids. All spectra are measured at MAS frequency ~6000 ± 3 Hz.

Solution-State NMR and TLC in this study confirm first that unbound lipids exist and further, were successfully removed from the sheds. Lipid components are similar in the toe and skin sheds (Figure 3b, Supplementary Figure S2 and Table 1). Solid-State NMR results on the adhesive toe pad sheds (^13^C CP/MAS, DP/MAS and ^1^H/MAS; Figure 4, 5a and 5b respectively) confirm the presence of lipids. As expected, lipids were also detected in skin sheds (Supplementary Figure S3a-b and Supplementary Figure S4). Upon extraction of unbound lipids^42^, the presence of the residual peak in the lipid region of the delipidized spectra indicates that there may be bound lipids present in the sheds^38^. Interestingly, major phospholipids found in the gecko footprints (PC and SM), are present in the toe shed extracts, consistent with the observation that geckos leave footprints on surfaces^15^.

Although we see sharp signatures for lipids in the ^13^C CP/MAS spectra, overall it seems the lipids are mobile compared to the rigid keratin proteins in the sheds. The appearance of a sharp lipid peak (33 ppm) in the ^13^C CP/MAS is due to the methylene group of the crystalline all-trans hydrocarbon chains present in general lipid structure^39^. Conversely, in ^13^C DP/MAS the liquid like trans/gauche conformation appears at 30–31 ppm, thus the sharp peak observed in the ^13^C DP/MAS at around 30 ppm (Table S1) is more strongly supported^39^. While the difference in mobility of keratin and lipids is intriguing, it is important to remember that the sheds from the toe pad are not exclusively comprised of setae; they are attached to several layers of epidermis. However, the setae account for ~65–70% of the total mass (Supplementary Text S2.1, 2.3).

**Figure 6:**
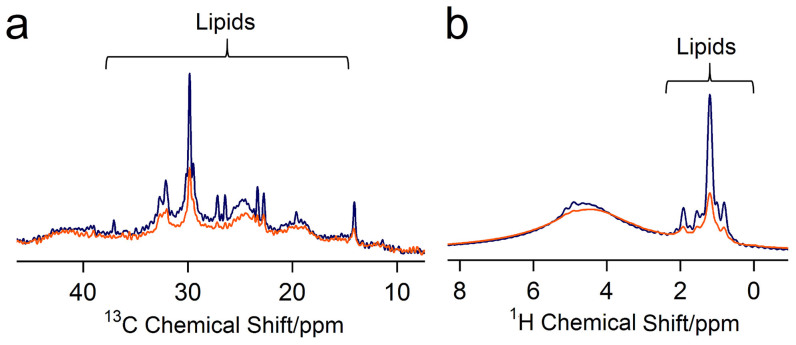
Comparing Pristine Epidermal (Toe and Skin) Sheds. Figure a and b are the direct polarization and proton magic angle spinning spectra respectively for pristine toe (blue) and pristine skin (orange) sheds. The nature of the lipid region differs in the toe and skin shed samples highlighting the difference in the lipid mobility between the two types of sheds from the gecko epidermis. All spectra are measured at MAS frequency ~6000 ± 3 Hz.

There are clear differences in lipid mobility between toe and non-adhesive skin (Figure 6a and Figure 6b respectively). Pristine toe sheds show sharper lipid peaks compared to the pristine skin, indicating that lipids are more mobile in the setae than the skin. Interestingly, another major difference between the skin and toe shed samples is seen in their response to delipidization. Visually, after delipidization clear differences are seen in the texture of the toe and skin sheds. While the toe sheds seem to be intact, the skin sheds seem to become rough and break after the treatment (small skin pieces are seen in the solvent mixture post treatment). Interestingly however, we do not see a similar behavior in the adhesive toe pad sheds. This contrasting behavior, in addition the difference in lipid mobility between toe and skin sheds may not be surprising as it is consistent with previous observations of the differential organization of lipids and keratin in the adhesive setae and in the skin of a gecko and an anole^14^. Indeed, recent analyses of specialized regions of epidermis in lepidosaurs like the adhesive setae of geckos^22^ or the ventral scales of snakes^53^, suggest that protein and lipid distribution may vary in response to functional roles of the epidermis. For example, even though an alpha and mesos layer may be conserved in structure and function across species and region of the epidermis, outer layers such as the beta and oberhautchen may differ in protein and lipid content organization^54^. Moreover, it may be important to distinguish how variation in protein-lipid interactions could be driven not only by function in the mature epidermis, but also in the development of the tissue itself.

**Figure 7:**
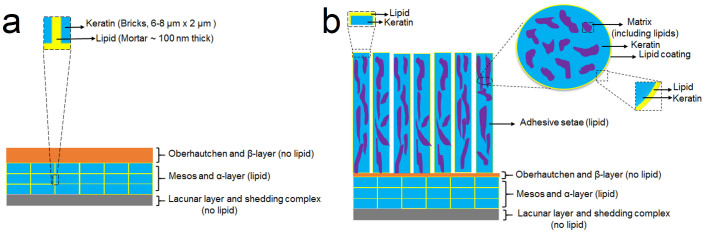
Lipid arrangement in skin and toe sheds. Figure a shows the layers of skin shed including the brick and mortar-based mesos and alpha layer rich in lipids. Figure b shows layers in the toe pad shed, where setae (lipid rich) are added to the skin layers described in Figure a. The setae likely contain lipids in the form of a thin coating (yellow), in the adhesive spatulae (not shown) and matrix. The matrix is a combination of lipids and unknown material (purple). The figures have not be drawn to scale and the spatula have not been included in the rendering of Figure b.

Our observations of different responses in NMR hints that there are differences in keratin and lipid associations (chemical or physical) in the two types of epidermal sheds. Past immunological and ultrastructural studies involving characterization of keratin shows the presence of two major beta proteins Ge-cprp-9 (cysteine rich) and Ge-gprp-6 (glycine rich), as well as alpha keratin proteins, Alfa1 and Alfa2 in the gecko setae^20^. Raman spectroscopy also confirmed the presence of alpha and beta keratins in the setae with primarily cysteine/phenylalanine/tyrosine signatures^24^. These signatures were detected in the ^13^C CP/MAS spectrum for the toe sheds (Figure 4, Supplementary Table S1). On the other hand, the non-adhesive skin is comprised of the proteins Ge-gprp-1, Ge-gprp-3, Ge-gprp-4, Ge-gprp-6, Ge-gprp-7 and Ge-gprp-8 with amino acids glycine, serine and proline being the most abundant^50^, all of which could be seen in ^13^C CP/MAS results for skin sheds (Supplementary Figure S3a, Supplementary Table S2). The ^13^C CP/MAS spectra for both toe and skin sheds show amino acid peaks dominating the spectra, indicating the rigid nature of the keratin. Our TLC and Solution State NMR results report that there is little to no difference in lipid composition between the adhesive toe pad sheds and the skin, yet clearly the keratin components do differ, specifically in the dominance of particular amino acids. It may be these differences that result in the difference in lipid mobility between toe pad shed and skin shed. Another possibility is the difference in the physical arrangement of lipids with the keratin in the toe and skin sheds. It is known that the lipids in the skin shed (brick and mortar layer) (Figure 7a) may be arranged in an orderly manner (orthorhombic or hexagonal)^55^, thus being less mobile. In contrast, lipids in the toe shed are also present in the setae as a part of the matrix^14^,^24^,^56^, surface coating^15^ and/or spatulae^15^ (Figure 7b). In these locations lipids are likely in a more disordered manner and are thus more mobile. We believe that the difference in lipid mobility in the skin shed and toe pad shed is either: 1. related to the chemical association of keratin components with lipids, where lipids in the toe pad sheds are more mobile by association with the cysteine and glycine rich keratin^22^, as opposed to the lipids in the skin shed which are associated with glycine, serine and proline rich keratin^50^ or 2. due to the difference in the physical association between the keratin and lipid components^55^. Clearly further investigation is necessary to fully understand this complex relationship but NMR results provide evidence that the setal structures on the gecko toe pad are not just morphologically specialized but also perhaps chemically specialized, where unbound lipids are weakly associated with the rigid keratin proteins. This hypothesis is further supported by the relatively easy and routine deposition of lipid footprints by the gecko as it moves across surfaces^15^.

Finally, we can use results above to compare proposed models for keratin-lipid association in the setae^15^. The toe sheds consist of outer keratinized setal hairs and several inner layers of cells that may contain lipids (Figure 7). The α-layer present underneath consists of “brick and mortar” pattern, where keratinocytes form the “bricks” and the lipids form the “mortar”^55^,^57^. Based on the amount of lipid extracted from the toe sheds and the dimensions of the bricks and mortar reported in previous publications, we can estimate that ~ 11 wt.% of the setal hairs are composed of lipids (detailed analysis is provided in Supplementary Text S2). The models^15^ where lipids are only present as an outer thin layer on setal hairs or as a major component in the spatula seem unlikely because the mass of lipid extracted is far greater than that predicted based on the amount of lipids extracted from the setal hairs (Supplementary Text S2.5). A more realistic model is the heterogeneous model^15^, where the lipids are distributed with the keratin throughout the setal hairs. However, the Solid-State NMR results suggest that spatial proximity of keratin and lipids has to be larger than 0.5 to 1 nm^58^, this is because after lipid extraction the protein peaks were unaffected. Therefore, a more realistic model of lipid distribution within the setae would be similar to the transmission electron microscopy (TEM) cross-section images published by Rizzo et al.^24^ and Huber et al.^56^ In this region of specialized epidermis, keratin is not organized into lamellar blocks as in coenocytes, but instead into long filaments of uncertain nano-construction^59^. Huber et al.^56^, showed that the darker-colored keratinized regions (69% by volume, 80–100 nm in diameter, and microns in length) are separated by lighter-colored ‘matrix’ region (31% by volume)^56^. The TEM images did not provide the chemical composition of the matrix and we propose that part of that matrix is composed of unbound lipids (almost 37% of the matrix based on the amount extracted, Text S2.5), as has been proposed for the lipid-keratin association in mammal stratum corneum^59^. A physical model illustrating the association of the lipids with keratin is shown in Figure 7B. In this model, we have included the possibility that the outer thin layer is still composed of lipids. In addition, it is also possible that there are bound lipids associated directly with keratin. Upon removal of unbound lipids, the structure of the setal hairs is still intact in contrast to the extraction of the lipids from the skin. In the case of skin, the extraction affects its physical integrity. Although further work is required to confirm this model organization, it is intriguing to consider a specific keratin and lipid architecture in the setae, perhaps for use as a specialized controlled wear component, where lipids are sacrificed at the adhesive interface by being more mobile than those same lipids in the non-adhesive skin.

In summary, we detected lipids in the adhesive setae of gecko toe pad sheds using NMR-based techniques. First, the sheds were delipidized to remove loosely bound surface lipids, the removal being evident in the NMR results. Additionally, the lipid extract was characterized using Thin Layer Chromatography and Solution-State NMR. Second, Solid-State NMR was used to investigate the association and dynamics of the lipid and keratin components of both toe and skin sheds. Analysis on the non-adhesive skin was primarily done to differentiate between the two types of material in the toe pad sheds (setae and underlying skin). Similar lipid associations were found in the toe pad shed and the non-adhesive skin shed but clear differences were seen in the dynamic behavior of the respective lipid regions. Lipids in the toe shed were more mobile than those in the skin sheds, suggesting that the specialized adhesive setae are chemically or physically structured differently than the rest of the epidermis. These findings have important implications for understanding the assembly of lipids and keratin in the adhesive setae as well as in fabrication of gecko-like adhesives using a mixture of materials. We also believe that the presence of lipids in multiple natural adhesive systems, ranging from barnacles^11^ to geckos^14^,^15^, highlights an important role of lipids in these systems, which needs to be more fully appreciated and investigated. Clearly our work here provides evidence that the lipid-keratin association in the specialized adhesive structures of the gecko is specific to those structures and thus may be relevant to their function.

## Methods

### Collection and preparation of samples of sheds

Freshly shed toe and skin molts were collected from *Gekko gecko* and preserved at −20°C. Precautions were taken to prevent the sheds from coming into contact with the hand while collecting (Supplementary Figure S1). The collected toe sheds were carefully examined and cut with a blade and a tweezer under an optical microscope to remove the skin surrounding the shed^60^. All procedures using live animals were approved by the University of Akron IACUC protocol 07-4G and were carried out in accordance with guidelines published by the Society for the Study of Amphibians and Reptiles (SSAR 2004).

### Lipid extraction

Pre-weighed samples of toe/skin sheds from *Gekko gecko* were treated with a solvent mixture for removal of unbound lipids. The samples were placed in 60 ml chloroform and methanol (Sigma Aldrich) mixtures successively (2:1, 1:1 and 1:2) for 2 hours. Each treatment was then repeated again for 1 hour. Thereafter the delipidized sheds were separated from the solvent mixture and dried in vacuum to remove traces of solvent. The solvent extract was collected and subjected to rotary evaporation under reduced pressure to procure the dried lipid^42^.

### Thin Layer Chromatography

The dried extracted lipid from the sheds (toe and skin) was dissolved in chloroform and applied to a 5 cm × 2 cm silica plate column with a micropipette. Lipid standards (1-palmitoyl-2-oleoyl-*sn*-glycero-3-phosphocholine (PC) and N-Nervonoyl-*D-erythro*-Sphingosylphosphorylcholine (SM), Avanti Lipids) dissolved in chloroform were also applied on the same plate. The plate was then dried in air for few minutes and developed in a small vial. A solvent mixture of chloroform-methanol-water (25:10:1, v/v/v) (Sigma Aldrich) (AOCS, Lipid Library) was used to develop the chromatograms and allowed to run through the plate for 10 minutes. After that, the plate was dried with a hair dryer and sprayed with either ninhydrin, 40% sulphuric acid or primuline (spot detection agents). The acid or ninhydrin sprayed plates were then heated at 110°C in an oven to char the lipids and observe the separated lipids as colored spots, while the primuline treated plates were observed under UV to view the spots^44^,^45^.

### Scanning Electron Microscopy

Images were taken using a JEOL JSM-7401F field emission scanning electron microscope at different magnifications. The pristine and delipidized toe/skin sheds were sputter coated with silver particles and were placed on the aluminum stubs lined with conductive carbon tape^60^.

### Sample preparation for Nuclear Magnetic Resonance (NMR) Spectroscopy

#### (a) Solid-State

Pristine or dried delipidized sheds (toe and skin separately) were weighed (~ 0.05 g) and packed in the 4 mm solid state rotor (Bruker). Teflon tape was inserted to pack the sample tightly.

#### (b) Solution-State

^1^H/^31^P NMR: The dried extracted lipid (from the toe and skin sheds separately) was dissolved in a 2:1 mixture of deuterated chloroform and deuterated methanol (Cambridge Isotope Lab.) for ^1^H NMR, or deuterated water (Cambridge Isotope Lab.) containing 250 mM sodium cholate (Alfa Aesar) and 5 mM EDTA (Calbiochem) for ^31^P NMR^49^. Samples were then packed in a 5 mm solution NMR tube for analysis.

### NMR Measurements

#### (a) Solid-State

All experiments were carried out with a Bruker AVANCE 300 MHz NMR equipped with a 4 mm double resonance VT CPMAS probe at 298 K. The ^1^H and ^13^C carrier frequencies were 300.1 and 75.6 MHz, respectively. The MAS rate was set to 6000 ± 3 Hz. The ^13^C chemical shift was referenced to the CH signal of adamantane (29.46 ppm) and ^1^H chemical shift with tetrakis(trimethylsilyl)silane (0.2 ppm) as an external reference. The 90° pulses for ^1^H and ^13^C were 4 μs while the recycle delay and contact time were 2 s and 2 ms, respectively. High-power Two Pulse Phase Modulation (TPPM) decoupling with field strength of 88 kHz was applied to the ^1^H channel during an acquisition time of 55 ms. ^13^C DPMAS spectra were obtained with a recycle delay of 15 s. ^1^H MAS spectra were obtained by a simple single pulse with a receiver delay of 6.5 µs and a recycle delay of 2 s.

#### (b) Solution State

^31^P and ^1^H NMR spectra were recorded at 313 K and 298 K respectively on a Varian INOVA 400 MHz spectrometer. Chemical shifts were recorded in ppm (δ) relative to 85% orthophosphoric acid (Phosphorus) and CDCl_3_ (Proton). ^31^P NMR spectra were recorded for 1648 scans with a 1 s delay using a 90° pulse width of 7.6 µs and an acquisition time of 1.6 s. ^1^H NMR spectra were recorded for 16 scans with a delay of 3 s, acquisition time of 1.6 s and a 90° pulse width of 9.75 µs.

## Supplementary Material

Supplementary Information Supplementary Information

## Figures and Tables

**Table 1:** Retention factors for extracted lipids from toe and skin sheds measured using Thin Layer Chromatography

Retention Factor (R_f_)				
Lipid	Literature Values	Toe Extract	Skin Extract	Standard Lipids
***Sphingomyelin(SM)***	0.11–0.15	0.12–0.15	0.11–0.16	0.14–0.15
***Phosphatidylcholine(PC)***	0.26–0.30	0.27–0.32	0.24–0.29	0.28–0.30
***Phosphatidylethanolamine(PE)***	0.50–0.54	0.53	0.53–0.55	N/A
***Non Polar Lipids (Glycerides, Fatty acids, Cholesterol)***	0.80–1	0.82–1	0.87–1	N/A
