# Supplementary material for: NMR spectroscopy reveals the presence and association of lipids and keratin in adhesive gecko setae
Source: Sci Rep. 2015 Apr 22;5:9594. doi: 10.1038/srep09594 (PMC5386106; doi:10.1038/srep09594)
Supplement: Supplementary Information [file srep09594-s1.pdf]

Supporting Information

NMR Spectroscopy reveals the presence and association of lipids and keratin in adhesive gecko setae

Dharamdeep Jain[#], Alyssa.Y.Stark[%], Peter H. Niewiarowski[%], Toshikazu Miyoshi[#], and Ali Dhinojwala[#]*

[#] Department of Polymer Science, The University of Akron, Akron, OH 44325-3909, USA.

[%] Department of Biology, Integrated Bioscience Program, The University of Akron, Akron, OH 44325-3908, USA.

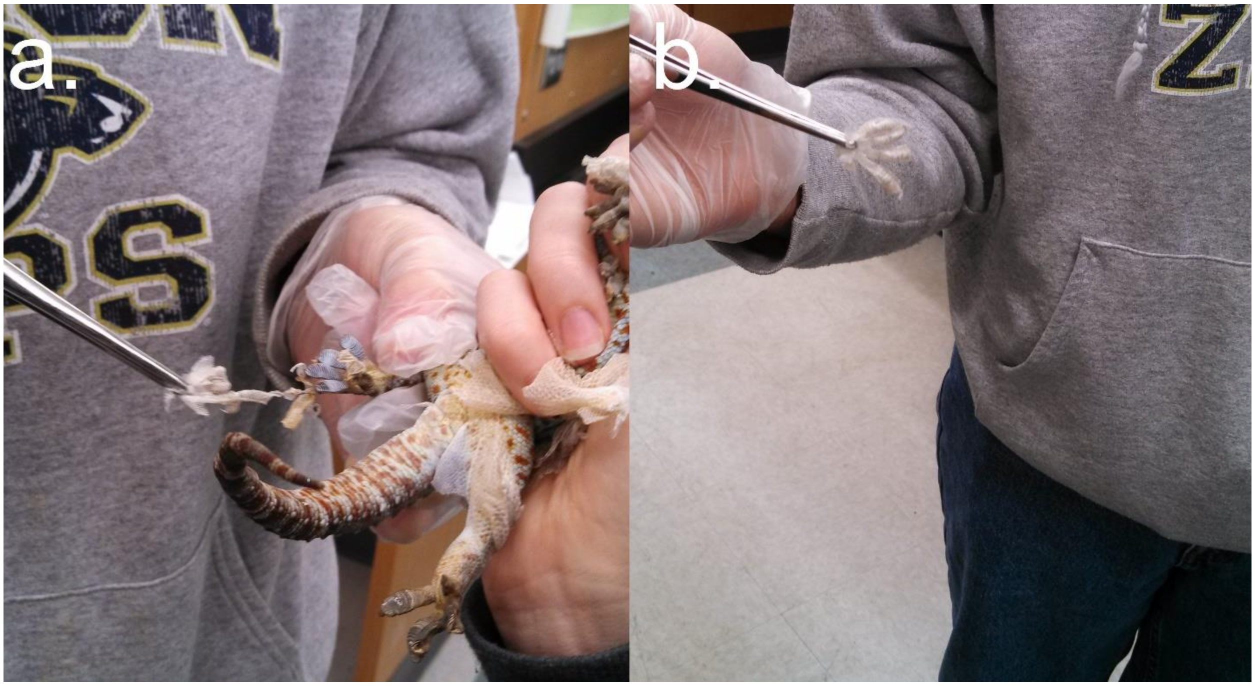

Figure S1.Collection of gecko sheds. *Figure a shows the removal of freshly molted gecko toe shed from Gekko gecko. The shed is carefully peeled with the help of tweezers and is not allowed to come in contact with bare hands. Figure b shows the removed shed from the toe region. The toe shed is accompanied with the non-adhesive skin which is isolated to get toe pad sheds.*

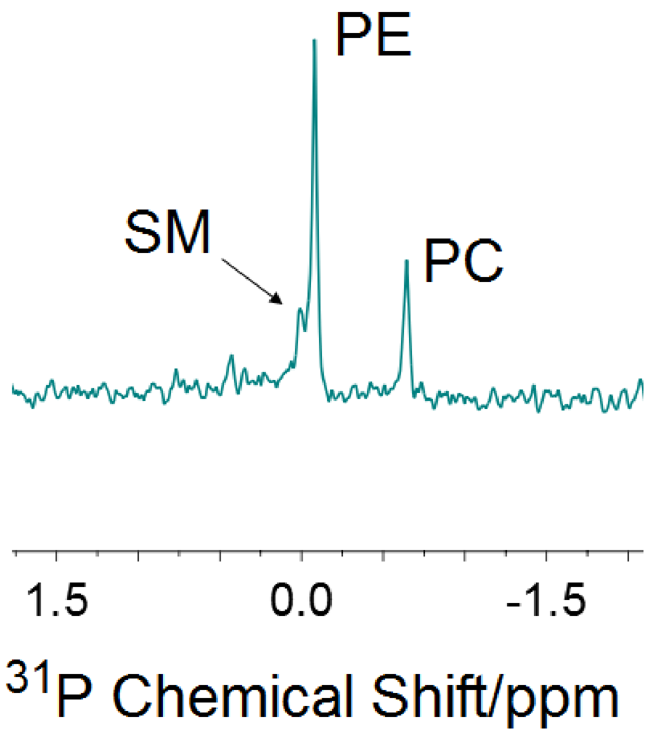

Figure S2. ^{31}P Solution NMR of lipid extracts from skin shed. *Figure shows the phospholipids detected in the skin shed extract using ^{31}P Solution NMR.*

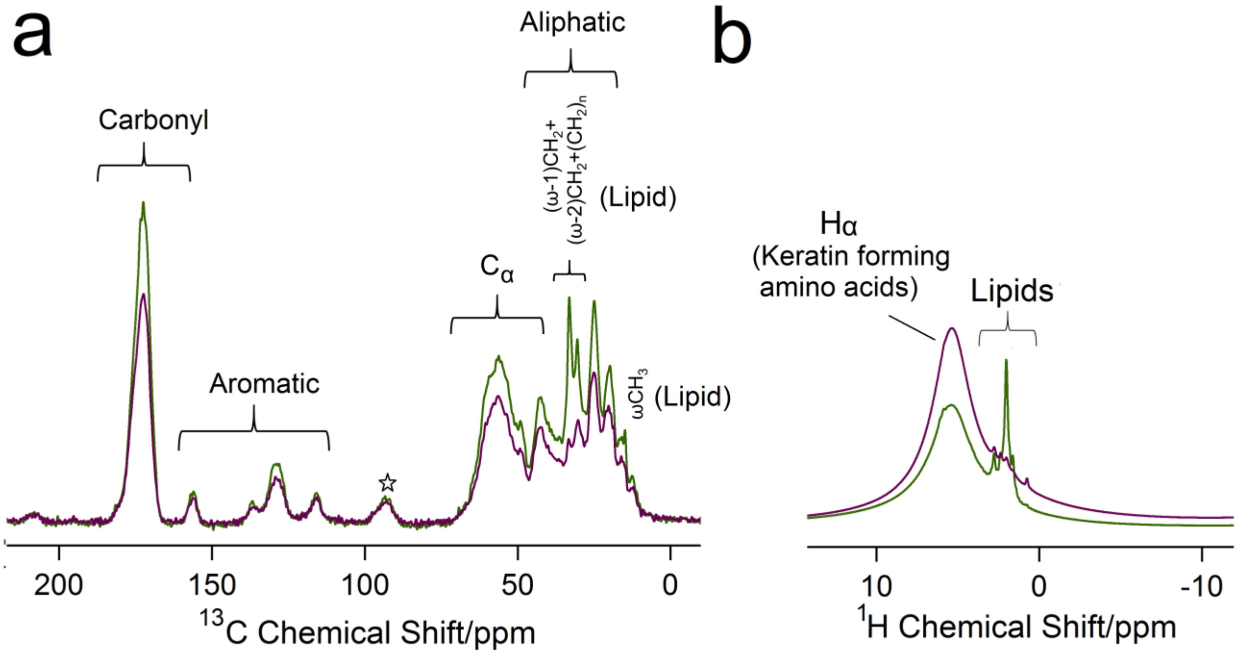

Figure S3. Cross Polarization/Magic Angle Spinning (CP/MAS) and ^{1}H Magic Angle Spinning (^{1}H/MAS) spectra for Pristine and Delipidized Skin Sheds. *Figure a shows the CP/MAS spectrum for the pristine (green) and delipidized (purple) for skin sheds. The lipid region lying in aliphatic range shows $(CH_2)_n$, (ω-1)CH_2, (ω-2)CH_2 and ωCH_3 signatures. The delipidized skin shed spectrum shows the removal of lipid (reduced peaks). The star labeled peak refers to the spinning sideband. Figure b is the ^{1}H/MAS spectrum for the pristine and delipidized skin sheds, showing the sharp lipid peak in the pristine sample which reduces after delipidization. All spectra are measured at MAS frequency ~6000±3 Hz.*

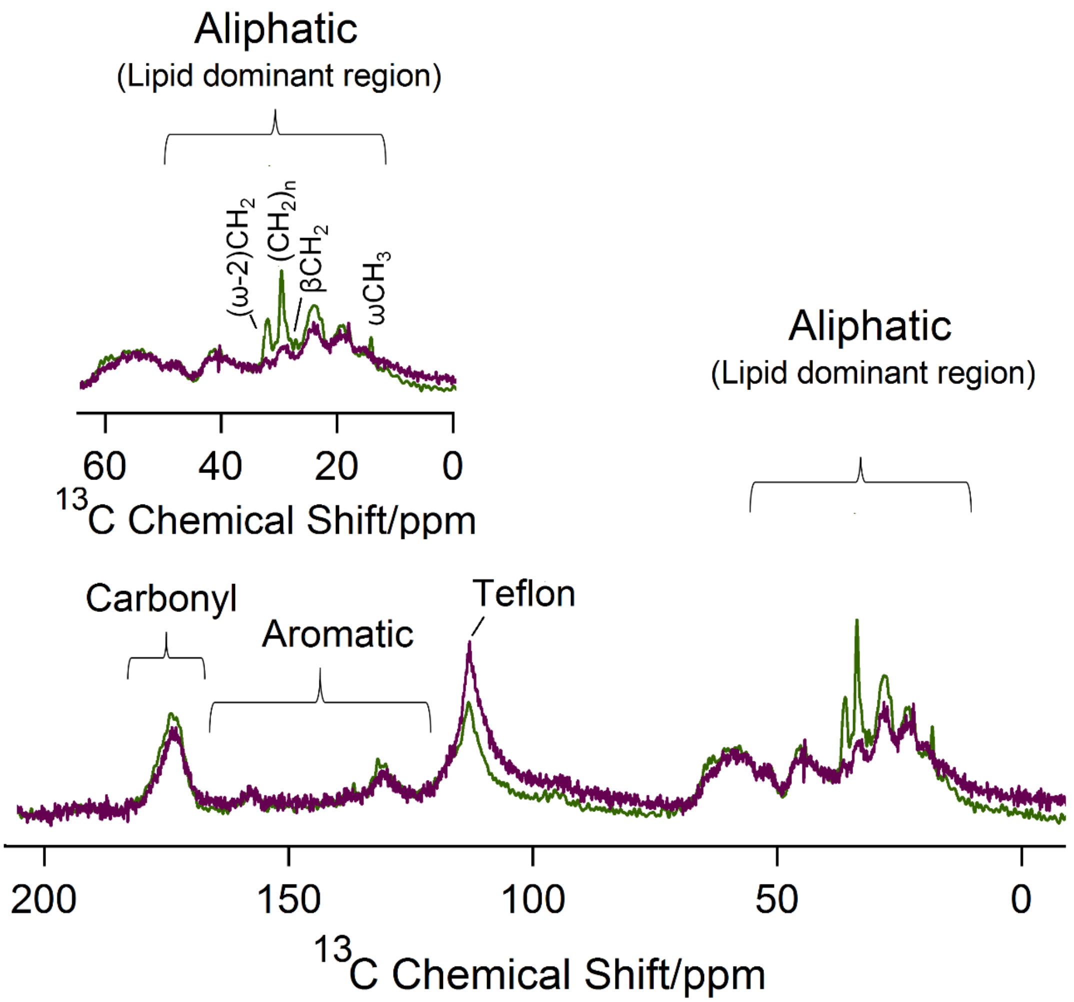

Figure S4.Direct Polarization Magic Angle Spinning (DP/MAS) for Pristine and Delipidized Skin Sheds. *Figure shows the DP/MAS spectrum for pristine (green) and delipidized (purple) skin sheds. The technique highlights the mobile components. Sharp signals dominate the aliphatic region that covers the lipid peaks. Inset is the enlarged aliphatic region (0-60 ppm) showing lipid signatures* $(CH_2)_n$, $(\omega\text{-}2)CH_2$, βCH_2 *and* ωCH_3. *Delipidized spectrum shows the reduction in lipid peaks. Teflon is used as the packing material and shows strong signal in DP/MAS. The spectra are measured at MAS frequency ~6000±3 Hz.*

Text S1. ***Solid State NMR (ssNMR)***

In general, solids with NMR active nuclei (spin quantum number ~1/2 such as ^{13}C,^{31}P, ^{15}N) show broad and structureless lineshapes due to magnetic anisotropic interactions such as dipolar interactions and chemical shift anisotropy (CSA). Unlike solution NMR, where sharp signals are seen due to rapidly isotropic motions, it becomes difficult to interpret the broad signals in solid state due to less mobility and its anisotropy. Hence high resolution spectra of dilute spins are obtained by incorporating Magic Angle Spinning (MAS) and dipolar decoupling with Cross Polarization (CP). MAS rotates the sample at 54.7^0 inclined from the magnetic field direction. Solid-State NMR for biological solids have been used extensively to get structural information such as chemical identity, molecular mobility, interatomic distances and conformations. The following are the basic details for the Solid-State NMR techniques used in the present study:

Direct Polarization/Magic Angle Spinning (DP/MAS)

DP/MAS (single pulse or Bloch decay) is based on direct excitation of the ^{13}C nuclei in the sample. The method highly relies on the spin-lattice relaxation time in the laboratory frame (T_1) of ^{13}C spins, which is in order of tens of seconds or minutes in solids. When repetition time for single time acquisition is set to a time longer than 5 times of T_1, DPMAS spectra provides quantitative information about signal heights. This technique is useful in observing the flexible/mobile regions in a sample.

Cross Polarization/Magic Angle Spinning (CP/MAS)

CP/MAS allows signal enhancement and much shorter acquisition times for observing rare nuclei like ^{13}C. This technique involves the transfer of magnetization from abundant spins like ^{1}H to rare spins such as ^{13}C, under what is known as the Hartmann-Hahn condition. Due to shorter relaxation times (in the range of ms) for abundant nuclei like ^{1}H, the acquisition times are less than DP/MAS. This technique is effective in recording signals from rigid regions of the sample.

High resolution Magic Angle Spinning Proton NMR (^{1}H/MAS)

The MAS coupled technique involves direct observation of abundant nuclei. It helps in probing mobile regions of the sample. The high mobility of the ^{1}H nuclei weakens the dipolar interactions leaving highly resolved signals (1).

Text S2 Calculations for testing the proposed models for keratin lipid associations (models described in Hsu PY et al. (2011) Direct evidence of phospholipids in gecko footprints and spatula-substrate contact interface detected using surface-sensitive spectroscopy. *J R Soc Interface* 9(69):657–664.)

In order to calculate the amount of lipid present in each of the proposed models, we want to first estimate the following parameters:

(a) Mass of hairs in toe pad shed (Text S2.1)

(b) Number of hairs in toe pad shed (Text S2.2)

(c) Mass of extracted lipid from toe pad shed (Text S2.3)

(d) Amount of lipids in skin shed (based on the known brick and mortar model) and toe pad shed (based on the brick and mortar model, mass of hairs in toe pad shed and experimentally extracted lipid from toe shed) (Text S2.4)

Text S2.1 Estimating mass of hairs in a toe pad shed

Measured mass of the toe shed = $2.9*10^{-3} \pm 2.0*10^{-4}$ g

Mass of setal hairs in the toe shed = N*ρ*l*b*h

where,

N is the number of rows of setae in toe shed

ρ is the density of keratin

l is the length of a row of hairs

b is the width of a row of hairs

h is the height of a row of hairs

N~15±2, l~ 0.25±0.06 cm, h~ 0.014±0.001 cm, b~ 0.030±0.003 cm

The dimensions (l, w) and N are measured from a toe shed image captured using an optical microscope. The value for h was calculated using an image published in elsewhere (2). ImageJ was used to measure all other parameters.

Considering the density of keratin (~ 1.28 g/cm^3 with a known range of ρ ~ 1.28-1.33 g/cc) (3), we can take the average values for all parameters and vary only one: "l" to get the mass of the setal hairs in the toe pad shed.

Mass of setal hairs = $1.9*10^{-3}$ g ± $0.4*10^{-3}$ g

Hence, the % of the whole toe pad shed that is comprised of setae alone is as follows:

(Mass of setal hairs / Mass of toe shed)*100 = 65 ± 12 %

The estimate suggests that setae contributes to the majority of the mass of a toe shed ~ 65%, while the non-adhesive skin is around ~35%.

Text S2.2 Estimating number of hairs in a toe pad shed

Dimensions of gecko setae = $r_{setae:}$ 2.5 µm and h_{setae}: 100 µm (4)

Mass of single setae = $\rho_{keratin}$* 3.14* r_{setae}^2*h_{setae} ($\rho_{keratin}$: 1.28 g/cm^3)

Mass of single setae = $2.55*10^{-9}$ g ± $0.1*10^{-9}$ g

As per earlier calculations (Text S2.1),

Number of setal hairs in experimental toe shed= Mass of hairs in toe shed/ Mass of single setae

$$\sim 7*10^5 \pm 2*10^5$$

Known number of hairs per foot pad of gecko ~ 10^3-10^6 (4,5)

Hence, the estimated value lies in the range published in the literature.

Text S2.3 Estimating mass of extracted lipid contributed by setae from a toe pad shed

Amount of lipid removed from toe shed ~ 10% of the toe shed mass (see main text)

Extracted amount = $2.9*10^{-4} \pm 2*10^{-5}$ g

Lipid extracted from setae ~ 65% of the total extracted amount from the toe shed (since ~65% is the amount of setae in toe shed and the rest is the non-adhesive skin)

Lipid contribution from the setae in a toe shed is = $65\%* 2.9*10^{-4}$ g

$= 1.8*10^{-4}$ g

Text S2.4 Estimating lipid present in the toe pad shed and skin shed

For Skin Shed (refer Figure 7a)

In the mesos and α-layer we consider "bricks" as keratin cylinders with a radius of 3 µm (diameter ~ 6-8 µm) and height 2 µm. Hence, the volume of the keratin cylinder is:

Volume of each keratin cylinder = $\pi*3^2*2 = 18\pi\ \mu m^3$

In the "brick and mortar" model we consider the “mortar" layer to be a 100 nm thick lipid layer between the keratin bricks (6).

In order to calculate the volume of a keratin brick and lipid layer, the new radius and height would be:

Radius = keratin brick + lipid layer = 3+ 50/1000 = 3.05 µm

Height = keratin brick + lipid layer = 2+ 100/1000 = 2.1 µm

where the effective thickness of lipid layer is taken to be 50 nm since it is shared between two keratin cylinders.

Hence, the volume of skin shed is = $\pi * 3.05^2 * 2.1 = 19.5\pi\ \mu m^3$

Therefore, the % of the total mass of the skin shed that is comprised of lipid alone (present in the mesos and α-layer) is as follows:

% Lipid in Skin Shed = (19.5π-18π/19.5π)*100 ~ 8%

For Toe pad shed (refer Figure 7b)

We know as per earlier calculations (Text S2.1), setal hairs comprise approximately ~65% of the mass of the toe shed and rest is the non-adhesive skin (including the mesos, α-layer, lacunar and shedding layers**).**

Also earlier estimates found that ~ 10% (main text) of the mass of the toe pad is removed by lipid extraction.

Thus, considering the amount of extracted lipid from toe shed, % contribution of lipid from the setal hairs (M) alone can be calculated as:

M*% of hairs in toe shed + % lipid from mesos and α-layer* % of non – adhesive skin in toe shed = 10%

Substituting the parameters,

M* 65% + 8%* 35% = 10%

Solving the equation for M, the % of lipid from the setal hairs alone is: M ~ 11%

% of lipid in setal hairs is ~ 11%

Text S2.5 Estimating lipid present in the setal hairs as per earlier models of lipid-keratin arrangement (as discussed in (7))

(a) *Homogeneous Model (Lipids coating the setal rods)*

Considering 2 nm thick layer of lipid coating each of the setae rods,

Mass of lipid coating each setae = $\rho_{lipid} * V_{lipid}$

$= 0.9\ g/cm^3 * [3.14*(2.502*10^{-4})^2*100.2*10^{-4} – 3.14*(2.5*10^{-4})^2*100*10^{-4}]\ cm^3$

$= 5.85*10^{-12}\ g$

Now, total number of setal hairs in toe shed is ~ $7*10^5$,

Hence, amount of lipid present as per homogeneous model ~ $4*10^{-6}$ g

(b) *Heterogeneous Model (Lipids forming spatulae)*

Dimensions of a spatula: L: 0.8 µm and d: 0.1 µm (8)

Mass of a single spatula = $\rho_{keratin}$* 3.14* $r_{spatula}^2$*$l_{spatula}$ = $8*10^{-15}$ g

Number of spatula per setae ~100-1000 (9)

Hence, total spatula in toe shed ~ 10^8 (Taking 1000 spatula per setae)

Mass of spatulae in toe shed or amount of lipid present considering lipid forming spatulae =

10^8* $8*10^{-15}$ g ~ $8*10^{-7}$ g

The results show that amount of lipid predicted to be present considering either of the models is less than the experimental results (~$1.8*10^{-4}$ g, Text S2.3), suggesting that lipids are not arranged with keratin like either of these proposed models in an exclusive manner. However, they may be present in combination of the models described. The 11% lipid (Text S2.4) present in the setae should be a combination of the two models described above, in addition to the presence of lipid in the 'matrix' material present within setae fibrils (Figure 7b)

According to the model described in (10,11), it is known that 69% (by volume) keratinized regions are present with 31% 'matrix'. Taking into consideration this distribution and the 11% lipid in setal hairs (Text S2.4), we can calculate lipid present in matrix (P), as follows:

% of matrix in setae* % lipid in matrix region + % of keratinized (non-matrix) in setae*% lipid in keratinized (non-matrix) region = % lipid found in setae

31% * P% + 69%* 0% = 11%

Hence, unbound lipid present in matrix (P) ~ 37%

In summary, the unbound lipid distribution in the setae (11%) is proposed to be a combination of models described above (Figure 7b).

Table S1. *Chemical Shifts (ppm) for signatures seen in Natural abundance CP/MAS, DP/MAS and ^{1}H/MAS for pristine toe pad sheds.*

Peak Assignments for Solid State NMR Results for Pristine Toe Shed					
Cross Polarization Magic Angle Spinning (CP/MAS)		*Direct Polarization Magic Angle Spinning (DP/MAS)*		*Proton Magic Angle Spinning (^{1}H/MAS)*	
Chemical Shift (ppm)	Assignment	Chemical Shift (ppm)	Assignment	Chemical Shift (ppm)	Assignment
172.6	-C=O	173	-C=O	3-6	H_α (Amino acids)
156.8	Tyr C_ζ/ Arg C_z	135	Aromatic/C=C	1.8-2.5	$\alpha/\beta CH_2$
136.4	Phe C_γ	130.3	Aromatic/C=C	1.1-1.4	$(CH_2)_n$, $(\omega\text{-}1)CH_2$, $(\omega\text{-}2)CH_2$
128.9	Phe $C_{\delta,\varepsilon,\zeta}$ /Tyr C_γ	125.8	Aromatic/C=C	0.7	ωCH_3
115.8	Tyr C_ε	50-60	C_α (amino acids except Gly)		
56.1	C_α (amino acids except Gly)	42	C_α (Gly)		
42.4	C_α (Gly)	37.9	αCH_2 (lipid)		
33.0	$(CH_2)_n$ (lipid)	32-33	$(\omega\text{-}2)CH_2$ (lipid)		
30.6	Val C_β /Pro C_β /$(CH_2)_n$ (lipid)	30.2-30.5	$(CH_2)_n$ (lipid)		
25.1	Cys C_β/Pro C_γ/Leu C_γ	25.2	βCH_2 (lipid)		
19.9	Val C_γ/Thr C_γ	24.0	N/A		
16.3	Ala C_β/Ile C_γ	23.4	$(\omega\text{-}1)CH_2$ (lipid)		
14.9	Ile C_γ/ωCH_3 (lipid)	20.3	N/A		
12.2	Ile C_δ	14.7	ωCH_3 (lipid)		

Table S2. *Chemical Shifts (ppm) for signatures seen in Natural abundance CP/MAS, DP/MAS and ^{1}H/MAS for pristine skin sheds.*

Peak Assignments for Solid State NMR Results for Pristine Skin Shed					
Cross Polarization Magic Angle Spinning (CP/MAS)		*Direct Polarization Magic Angle Spinning (DP/MAS)*		*Proton Magic Angle Spinning (^{1}H/MAS)*	
**Chemical Shift (ppm)**	**Assignment**	**Chemical Shift (ppm)**	**Assignment**	**Chemical Shift (ppm)**	**Assignment**
172.7	-C=O	173	-C=O	3-6	$H_α$ (Amino acids)
156.8	Tyr $C_ζ$/ Arg C_z	130	Aromatic/C=C	1.8-2.5	α/βCH_2
136.4	Phe $C_γ$	50-60	$C_α$ (amino acids except Gly)	1.1-1.4	$(CH_2)_n$, (ω-1)CH_2, (ω-2)CH_2
128.9	Phe $C_{δ,ε,ζ}$ /Tyr $C_γ$	42	$C_α$ (Gly)	0.7	ωCH_3
116.1	Tyr C_e	33.1	(ω-2)CH_2 (lipid)		
50-60	$C_α$ (amino acids except Gly)	30.5	$(CH_2)_n$ (lipid)		
42.7	$C_α$ (Gly)	24.3	N/A		
33.1	$(CH_2)_n$ (lipid)	20.0	N/A		
30.4	Val $C_β$/Pro $C_β$/$(CH_2)_n$ (lipid)	14.7	ωCH_3 (lipid)		
24.9	Cys $C_β$/Pro $C_γ$/Leu $C_γ$				
20.3	Val $C_γ$/Thr $C_γ$				
16.3	Ala $C_β$/Ile $C_γ$				
14.7	Ile $C_γ$/ωCH_3 (lipid)				
12.1	Ile $C_δ$				

References

1. Saito, H., Ando, I. & Naito, A. [Solid State NMR Approach] *Solid State NMR Spectroscopy for Biopolymers: Principles and Applications* [1-15] (Springer, 2006)

2. Gillies, A. G. *et al.* Gecko toe and lamellar shear adhesion on macroscopic, engineered rough surfaces. *J. Exp. Biol.* **217,** 283–289 (2014).

3. Mason, P. Density and structure of alpha-keratin. *Nature* **197,** 179-180 (1963).

4. Autumn, K. & Peattie, A. M. Mechanisms of adhesion in geckos. *Integr. Comp. Biol.* **42,** 1081–1090 (2002).

5. Autumn, K. *et al.* Dynamics of geckos running vertically. *J. Exp. Biol.* **209,** 260–72 (2006).

6. Bouwstra, J. A., Honeywell-nguyen, P. L. & Gooris, G. S. Structure of the skin barrier and its modulation by vesicular formulations. *Prog. Lipid Res.* **42,** 1–36 (2003).

7. Hsu, P. Y. *et al.* Direct evidence of phospholipids in gecko footprints and spatula-substrate contact interface detected using surface-sensitive spectroscopy. *J. R. Soc., Interface* **9,** 657–664 (2011).

8. Tian, Y. *et al.* Adhesion and friction in gecko toe attachment and detachment. *Proc. Natl. Acad. Sci. U S A* **103,** 19320–19325 (2006).

9. Huber, G., Gorb, S. N., Spolenak, R. & Arzt, E. Resolving the nanoscale adhesion of individual gecko spatulae by atomic force microscopy. *Biol. Lett.* **1,** 2–4 (2005).

10. Huber, G., Orso, S., Spolenak, R., Wegst, U. G., Enders, S., Gorb, S. N., & Arzt, E. Mechanical properties of a single gecko seta. *Inter. J. Mater. Res.* **99,** 1113–1118 (2008).

11. Rizzo, N. W. *et al.* Characterization of the structure and composition of gecko adhesive setae. *J. R. Soc. Interface* **3,** 441–451 (2006).
